# Full Step Cycle Kinematic and Kinetic Comparison of Barefoot Walking and a Traditional Shoe Walking in Healthy Youth: Insights for Barefoot Technology

**DOI:** 10.1155/2017/2638908

**Published:** 2017-11-07

**Authors:** Yi Xu, Qinghua Hou, Chuhuai Wang, Andrew J. Sellers, Travis Simpson, Bradford C. Bennett, Shawn D. Russell

**Affiliations:** ^1^Department of Rehabilitation Medicine, The 1st Affiliated Hospital, Sun Yat-sen University, Guangzhou 510080, China; ^2^Department of Orthopaedic Surgery and Mechanical Engineering, Motion Analysis & Motor Performance Laboratory, University of Virginia, Charlottesville, VA 22903, USA; ^3^Department of Neurology, The Seventh Affiliated Hospital, Sun Yat-Sen University, Shenzhen 518107, China; ^4^CDR, MC USN, Department of Radiology, Naval Medical Center Portsmouth, Portsmouth, VA 23708, USA; ^5^Department of Kinesiology, California State University East Bay, Hayward, CA 94542, USA

## Abstract

**Objective:**

Barefoot technology shoes are becoming increasingly popular, yet modifications are still needed. The present study aims to gain valuable insights by comparing barefoot walking to neutral shoe walking in a healthy youth population.

**Methods:**

28 healthy university students (22 females and 6 males) were recruited to walk on a 10-meter walkway both barefoot and in neutral running shoes at their comfortable walking speed. Full step cycle kinematic and kinetic data were collected using an 8-camera motion capture system.

**Results:**

In the early stance phase, the knee extension moment (MK1), the first peak absorbed joint power at the knee joint (PK1), and the flexion angle of knee/dorsiflexion angle of the ankle were significantly reduced when walking in neutral running shoes. However, in the late stance, barefoot walking resulted in decreased hip joint flexion moment (MH2), second peak extension knee moment (MK3), hip flexors absorbed power (PH2), hip flexors generated power (PH3), second peak absorbed power by knee flexors (PK2), and second peak anterior-posterior component of joint force at the hip (APFH2), knee (APFK2), and ankle (APFA2).

**Conclusions:**

These results indicate that it should be cautious to discard conventional elements from future running shoe designs and rush to embrace the barefoot technology fashion.

## 1. Introduction

The description of the Tarahumara Indian running tribe in the book *Born to Run* by Christopher McDougall has inspired a renewed enthusiasm for barefoot running and led to an ongoing movement to simplify the inner structure of shoes and thus has boosted the development of Minimalist Barefoot Technology (MBFT) shoes, which are extremely flexible and have low heel to toe drop, weight, and stack height, that is, having little to no cushioning [1].

However, the effectiveness of MBFT shoes is somewhat ambiguous. There are accumulating positive studies that favor the MBFT shoes. In a group of elderly women with knee osteoarthritis, Trombini-Souza et al. [2] noticed that MBFT shoes possess the capability of reducing joint moment impulse in women with knee osteoarthritis and enhancing trunk muscle activities in a healthy population [3]. In the meantime, negative effects of MBFT have also been noticed. Examples included studies that found higher vertical forces of MBFT shoe walking [4]over walking with neutral running shoes or walking barefoot and that running in MBFT shoes increased the loads to the lower extremity, knee flexion/dorsiflexion angle, average vertical component of ground reaction force [5], and Achilles tendon force [6] as compared to running with neutral running shoes. These bring into question that modern nonhabitually barefoot adults could adapt to current MBFT shoes well. Actually, modifying the construction of MBFT shoe to make it better fit to modern nonhabitually barefoot adults has attracted a big amount of research interest in recent years [7, 8]. Investigation into the pros and cons of barefoot and shod walking/running is thus still of value with the goal of further optimizing barefoot technology.

In running studies, efforts to uncover the role of footwear are usually complicated by individual gait patterns (heel versus forefoot strike). In contrast, when people normally walk, heel strike is the only strike pattern typically seen [9]. The present study therefore aims to compare the joint angles, moments, powers, and forces (including vertical component and anterior-posterior component) through the whole step cycle in a healthy youth population, barefoot walking and walking in a widely used neutral running shoe, which has conventional design of heel lift and forefoot rocker, but no lateral posting used to stabilize the hind foot. We hypothesize that barefoot walking and shod walking might have respective merits in different phases of the step cycle; however, as people of modern society would seldom take barefoot as routine, our goal of the full step cycle comparison between them is to get valuable insights for the future design of MBFT shoes.

## 2. Subjects and Methods

30 healthy university students, who were neurologically healthy confirmed by on-field examination by a PM&R physician (Y. X.), volunteered in the present study. All the tests and analysis were performed in the Motion Analysis & Motor Performance Laboratory at the University of Virginia (UVa), and all procedures were approved by the Human Investigation Committee of UVA (HSR number: 16853). Consent was obtained from all the subjects enrolled.

When tested, enrolled subjects walked both barefoot and in neutral running shoes (Brooks^©^, Radius 06) along a 10-meter walkway, wearing a plug-in-gait full body 37-marker set (Vicon, Oxford, UK). The order of walking conditions tested was randomly decided by coin flipping. An 8-camera Vicon Motion Analysis System (Vicon, Oxford, UK) was used to collect 3-D kinematic data, and the data collecting frequency was 120 Hz. Simultaneously, ground reaction forces were collected via four in-ground force plates (Kistler, Switzerland, and Betec, OH) at 1080 Hz.

All subjects were required to walk on the walkway at least five times before each individual's test trial to acclimate to the footwear. During the test trial, subjects were instructed to walk at his/her self-selected comfortable walking speed (CWS). Yet, to make walking speed comparable in the two tested conditions, only trials with similar walking speeds were chosen from both tested conditions, at least five successful trials were recorded for each subject. A 3–5 min break was instituted for recovery between each trial.

With the Vicon Body Builder software, spatial-temporal parameters (walking speed, cadence, and step length), contact reaction forces and joint angles, joint moments (extension/flexion), and joint powers (generated/absorbed) in sagittal motion plane of the whole step cycle, which was designated by manually identifying the heel-contact and toe-off points, were extracted by means of inverse dynamics and analysis.

The peak values of kinematic and kinetic variables were computed with the definition and methodology of Eng and Winter [10], and the calculation of joint moment and joint power were normalized to the subject's body mass (kg).

1. At the hip joint
  - Power 1 of hip (PH1): the energy generated by the hip extensors
  - Power 2 of hip (PH2): the energy absorbed by the hip flexors
  - Power 3 of hip (PH3): the energy generated by the hip flexors
  - Moment 1 of hip (MH1): the peak hip extension moment at the early stance phase
  - Moment 2 of hip (MH2): the peak hip flexion moment
  - Moment 3 of hip (MH3): the peak hip extension moment at the late stance phase
2. At the knee joint
  - Power 1 of knee (PK1): the first peak energy absorbed by the knee joint
  - Power 2 of knee (PK2): the second peak energy absorbed by the knee joint
  - Moment 1 of knee (MK1): the peak knee extension moment at the early stance phase
  - Moment 2 of knee (MK2): the peak knee flexion moment
  - Moment 3 of knee (MK3): the peak knee extension moment at the late stance phase
3. At the ankle joint
  - Power 1 of ankle (PA1): the energy absorbed by the ankle joint
  - Power 2 of ankle (PA2): the energy generated by the ankle joint
  - Moment 1 of ankle (MA1): the peak ankle extension moment
  - Moment 2 of ankle (MA2): the peak ankle flexion moment

For contact reaction force, the vertical force 1/2 of the hip (VFH1/VFH2), vertical force 1/2 of the knee (VFK1/VFK2), and vertical force 1/2 of the ankle (VFA1/VFA2) each represents the landing/push-off activity as signified by the first/second peak of the vertical component of contact reaction force of the hip, knee, and ankle joint, respectively. Additionally, the anterior-posterior (A-P) force 1/2 of the hip (APFH1/APFH2), A-P force 1/2 of the knee (APFK1/APFK2), and A-P force 1/2 of the ankle (APFA1/APFA1) each represents the first/second peak A-P component of contact reaction force of the hip, knee, and ankle joint, respectively.

Matched sample Student *t*-tests were used to assess the differences in tested variables between the two conditions, and the normality of data was confirmed by Kolmogorov-Smirnov test. *p* < 0.05 was set as the criteria for statistical significance.

## 3. Results

Data of two subjects were incomplete and excluded. Thus, the present study had 28 enrolled volunteers in total, 22 females and 6 males. The demographic data of these volunteers were as follows: age: 20.1 ± 0.8 years; mass: 64.2 ± 9.0 kg; height: 167.5 ± 5.8 cm; and body mass index: 23.0 ± 3.7 kg/m^2^. Statistics analysis of spatial-temporal variables suggested that there were no significant differences in walking speed, cadence, step length, or stride length between the two conditions (Table 1).

**Figure 1:**
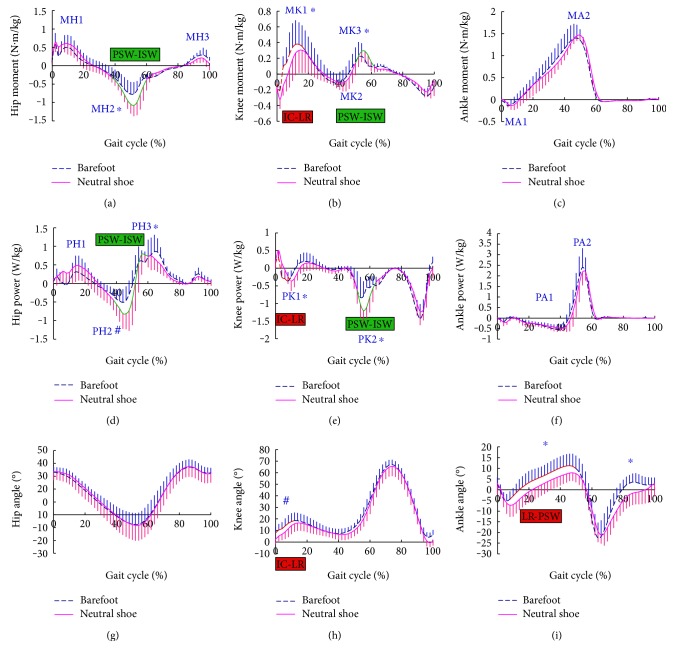
Joint moment (a, b, c), joint power (d, e, f), and joint angle (g, h, i) at the hip (a, d, g), knee (b, e, h), and ankle (c, f, i) in the sagittal plane during the gait cycle. On the curve of joint moment, the extension moment is graphed as a positive value above the horizontal axis, while the flexion moment is graphed as negative values below the horizontal axis. On the curve of joint power, generated power was graphed as a positive value above the horizontal axis, while absorbed power was graphed as a negative value below the horizontal axis. Curves show the mean value and one standard deviation for walking barefoot (blue dashed line) and neutral shoe (pink solid line), the value that is of significant difference was highlighted in red (for the duration from initial contact phase (IC) to loading response phase (LR)) and in green (for the duration from preswing phase (PSW) to initial swing phase (ISW)). ^∗^Points of significant differences and ^#^points of very significant differences are marked on the curve.

**Figure 2:**
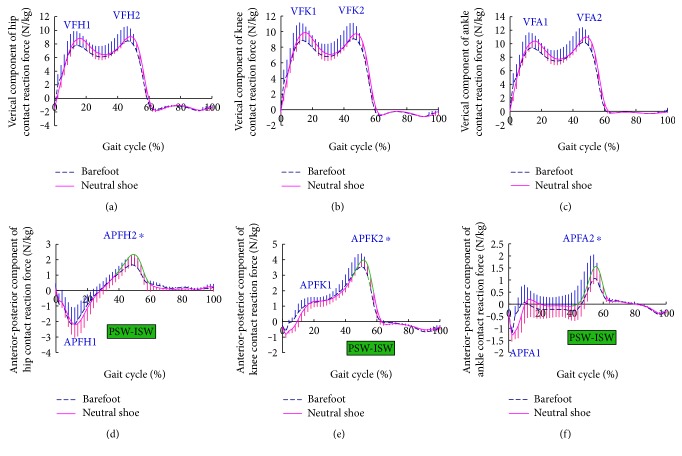
Vertical component of contact reaction force (a, b, c) and anterior-posterior component of contact reaction force (d, e, f) at the hip (a, d), knee (b, e), and ankle (c, f) in the sagittal plane during the gait cycle. Curves show the mean value and one standard deviation for walking barefoot (blue dashed line) and neutral shoe (pink solid line), the value that is of significant difference was highlighted in green (for the duration from preswing phase (PSW) to initial swing phase (ISW)). ^∗^Points of significant differences are marked on the curve.

Walking in neutral shoes attenuated the flexion angle of the knee at the early stance phase from 17.9 ± 7.14° (barefoot) to 14.43 ± 7.50° (in neutral shoes, *p* = 0.005), and the dorsiflexion angle at the ankle joint from 11.45 ± 5.56° (barefoot) to 8.06 ± 3.87° (in neutral shoes, *p* = 0.030), when compared with walking barefoot (Figures 1(g)–1(i)). Whereas no significant difference of ground reaction force was noticed at the early stage of the step cycle between the two conditions, as indicated by the values of VFH1, VFK1, VFA1, APFH1, APFK1, and APFA1 (Figures 2(a)–2(c)).

Compared to walking barefoot, walking in neutral shoes significantly reduced MK1 and PK1 in the sagittal motion plane at the knee joint [created between the initial contact phase (IC) to loading response phase (LR), i.e., in the early stance phase of gait cycle] (MK1: in neutral shoes 0.25 ± 0.20 N·m/kg versus barefoot 0.38 ± 0.29 N·m/kg, *p* = 0.038; PK1: in neutral shoes −0.18 ± 0.17 W/kg versus barefoot −0.34 ± 0.25 W/kg, *p* = 0.048), and no significant difference was discovered in other joint moments or joint powers in the early stance phase at the hip, knee, or ankle joints (Figures 1(a)–1(f)).

Compared to walking in neutral shoes, no significant difference in contact reaction force was noticed in the late stance phase of step cycle between the two conditions, as measured by VFH2, VFK2, and VFA2. Whereas, the APFH2, APFK2, and APFA2 from PSW to ISW (i.e., the late stance phase) of walking barefoot were markedly reduced (APFH2: in neutral shoes 2.20 ± 0.61 N/kg versus barefoot: 1.63 ± 0.49 N/kg, *p* = 0.032; APFK2: in neutral shoes 3.95 ± 0.46 N/kg versus barefoot 3.43 ± 0.77 N/kg, *p* = 0.046; APFA2: in neutral shoes 1.45 ± 0.56 N/kg versus barefoot 1.01 ± 0.71 N/kg, *p* = 0.027). Additionally, no significant difference was demonstrated in flexion and extension angles of the lower limb joints between the two conditions at the late stance phase (Figures 2(d)–2(f)).

From the preswing phase (PSW) to the initial swing phase (ISW) of the gait cycle (i.e., the late stance phase at which tested subjects are ready to push off), the difference in the value of joint moments reappeared. At this stage, MH2 at the hip joint was decreased markedly by walking barefoot compared to walking in neutral shoes (MH2: in neutral shoes −0.76 ± 0.32 N·m/kg versus barefoot −1.06 ± 0.31 N·m/kg, *p* = 0.042). Similarly, MK3 at the knee joint was reduced significantly by walking barefoot (MK3: in neutral shoes 0.30 ± 0.15 N·m/kg versus barefoot 0.18 ± 0.11 N·m/kg, *p* = 0.048). Also, at this stage of gait cycle, the PH2 and PH3 of walking barefoot were significantly lower than those of walking in neutral shoes (PH2: in neutral shoes −0.84 ± 0.44 W/kg versus barefoot −0.46 ± 0.38 W/kg, *p* = 0.009; PH3: in neutral shoes 0.77 ± 0.21 W/kg versus barefoot 0.56 ± 0.30 W/kg, *p* = 0.038). Additionally, the PK2 of walking barefoot was markedly lower than that of walking in neutral shoes (PK2: in neutral shoes −1.18 ± 0.31 W/kg versus barefoot −0.76 ± 0.41 W/kg, *p* = 0.026) (Figures 1(a)–1(f)).

## 4. Discussion

In the present study, it is noticed that loads (joint power and joint moment) and joint angles in the sagittal motion plane of the lower extremities in young healthy volunteers were reduced by walking with a neutral shoe at the early stance phase, while loads and anterior-posterior component of contact reaction force in sagittal motion plane at the late stance phase were augmented, as compared to walking barefoot.

Our finding that neutral shoe decreases the contact loads at the early stance is consistent with previous research of Yeow et al. [11]. Owing to their soft and viscoelastic characteristics, insole, heel pad, and wedge of the shoe can effectively cushion the contact loading and reduce heel pressure at initial contact [12, 13]. Additionally, according to earlier studies, these accessories of traditional shoes tend to shift the walker's center of mass (COM) anteriorly during walking [14–16], and in the present study, we notice that neutral running shoe walking tends to alleviate joint movement at the knee and ankle level. These effects may in turn alter limb alignment at heel landing, which is closely related to load and pressure distribution [17, 18], and herein help to dissipate the landing load further [19].

In the present study, barefoot walking significantly reduces joint moments and power of joint knee/hip in the late stance phase. Furthermore, barefoot walking is associated with a marked reduction of the A-P component of lower limb joints' contact reaction force in the hip, knee, and ankle. In a step cycle, the first peak of the A-P component of contact reaction force reflects the braking activities of landing, while the second peak reflects the activities of push-off (propulsion force) [20]. Furthermore, Turns et al. [21] have demonstrated that during steady-state walking, if the walking speed is equal, the net braking and propulsive impulses should be approximately the same. It then follows that being barefoot might facilitate push-off in walking, as a smaller A-P component of the contact reaction force would indicate a less muscular force which the lower limbs need to generate to achieve push-off. This finding is consistent with the conclusions of Zhang et al. [22].

The above findings indicate that barefoot walking is not overwhelmingly superior to shod walking, at least not during the whole gait cycle. At heel contact during human walks, the contact reaction force that can be as high as several times of body weight [23] goes firstly and directly to the heel, as human walking is usually executed in a heel strike pattern, though some contact reaction forces can be dissipated by the deformation of the soft tissue pad of the foot. However, the force dissipating capability of human heel pad, as Wearing et al. [24] has demonstrated, operates close to its pain threshold during barefoot walking and probably has no room for accidental raise of contact reaction force. As walking is such a common daily repetitive activity, a relatively small decrease in force dissipation over each step may significantly add to the cumulative loading on the lower limb's joints over time. Thus, our finding that shod walking decreases joint moment/power over barefoot walking, though small and limited to a few joints and variables, is likely still of value in the prevention of lower extremity injury. In fact, a variety of observations have noticed the injuries caused by MBFT shoes running, no matter the runners are new or habitual to the MBFT shoes [25–27] or have reported that some injury-decreased effects of MBFT shoes might be only a manifestation of the redistribution of mechanical work after MBFT transition [6, 28]. This has been further confirmed in a very recently randomized control trial by Fuller et al. [29], in which the authors randomly allocated 61 trained, habitual rearfoot footfall runners to either MBFT shoes or conventional shoes, and they found out that after 26 weeks of regular running, there are 11 of 30 runners sustained an injury in conventional shoes, while this number in the MBFT shoes group is 16/30, the hazard ratio is 1.64, and 95% confidence interval is 0.63–4.27. Though some previous authors would like to emphasize the strike-pattern modifying capacity of the MBFT shoes, that is, MBFT shoe tends to shift the rear foot strike pattern to forefoot strike pattern, which is believed to be capable of facilitating injury protection [30]. However, there are also evidences which demonstrated that education for assuring the strike pattern actually would not do much help to for the injury-decreased effect MBFT shoes [31, 32]. Along with these previous evidences, the above findings of the present study indicate that it should be cautious to discard conventional from future running shoe designs and rush to embrace the minimalism fashion.

While at the late stance phase during human walks, lower limb joints initially flex and then extend for push-off, as a result of muscular loading and unloading. It then makes sense that the integrated features of elastic energy storage and release of footwear might add to the efficacy of this procedure [33]; the characteristics of low mass and elastic and the least constraint of movement of barefoot walking might be the possible attributions [34] for the relatively better performance of barefoot in the late stance phase observed in the present study. The better performance of barefoot walking in the late stance phase might be a clue of value for future MBFT shoe design.

### 4.1. Limitations

Our study has several limitations. Firstly, all of the subjects are healthy, young, and predominantly female; thus, the results of this study may not be generalizable. Secondly, we did not give the subjects a familiarization period with the new footwear (either barefoot or traditional shoe). Therefore, our observations should only be interpreted as the acute kinetics or acute kinematic effects of walking barefoot or walking in a traditional shoe. Lastly, though the present study has carefully conducted a full step cycle comparison, it is only a primary study and has not tested any specific parameters of barefoot technology shoe design such as sole thickness, materials, insole composition, or shoe shaft construction, which all need to be investigated further.

## 5. Conclusions

The present study demonstrates that walking with traditional neutral running shoes reduces the loads and joint angles of the lower extremity significantly and more effectively than walking barefoot at the early stance phase, suggesting it should be cautious to discard conventional elements from future running shoe designs and rush to embrace the barefoot technology fashion.

## Figures and Tables

**Table 1:** Comparison of the spatial-temporal variables between two tested conditions (mean ± SD).

	Barefoot walking	Neutral shoe walking	*p* value
Cadence (step/min)	113.01 ± 4.99	112.16 ± 5.13	0.366
Walking velocity (m/s)	1.26 ± 0.12	1.28 ± 0.11	0.361
Stride length (m)	1.37 ± 0.08	1.34 ± 0.06	0.085

## Additional Points

CDR Sellers is a military service member. This work was prepared as part of Bradford C. Bennett's official duties. Title 17 U.S.C. 105 provides that “copyright protection under this title is not available for any work of the United States Government.” Title 17 U.S.C. 101 defines a United States Government work as a work prepared by a military service member or employee of the United States Government as part of that person's official duties.
